# Less Is More: Risk Factors and Survival Outcomes of Overtreatment for Early‐Stage Colorectal Cancer

**DOI:** 10.1002/jso.70028

**Published:** 2025-07-05

**Authors:** Daniel R. S. Habib, Matthew Shou, James L. Rogers, Kevin Sun, Chen Chia Wang, Aimal Khan

**Affiliations:** ^1^ Vanderbilt University School of Medicine Nashville Tennessee USA; ^2^ Department of Surgery, Section of Surgical Sciences Vanderbilt University Medical Center Nashville Tennessee USA

**Keywords:** adjuvant therapy, colon cancer, overall survival, overtreatment, rectal cancer, surgery

## Abstract

**Background and Objectives:**

After cT1‐2N0M0 colorectal cancer (CRC) definitive resection (colectomy/proctectomy) without pathologic upstaging, only observation is recommended given the lack of benefit from adjuvant treatment, which would constitute overtreatment. This study aims to determine risk factors and overall survival (OS) associated with overtreatment in early‐stage CRC.

**Methods:**

This National Cancer Database study included cT1‐T2N0M0 CRC patients who underwent definitive resection between 2010 and 2020. Multivariable logistic regressions were performed to assess overtreatment risk factors. After propensity‐matching, Kaplan–Meier survival analyses and multivariable Cox proportional‐hazards analyses were performed to assess the association of overtreatment with OS.

**Results:**

Of 22 875 colon cancer and 4198 rectal cancer cases, 144 (0.6%) and 82 (2.0%) were overtreated, respectively. Colon cancer overtreatment was associated with younger age (aOR = 0.96, 95% CI = 0.95–0.98), Black race (aOR = 1.94, 95% CI = 1.26‐2.99), and pT2 vs. pT1 (aOR = 1.66, 95% CI = 1.19–2.33). Rectal cancer overtreatment was associated with pT2 (aOR = 2.58, 95% CI = 1.59–4.19), poor/undifferentiated grade (aOR = 2.61, 95% CI = 1.44–4.76), and high‐risk histology (aOR = 3.20, 95% CI = 1.22–8.40). In the propensity‐matched cohorts, overtreatment was associated with worse OS for colon (HR = 1.40, 95% CI = 1.01–1.93) but not rectal cancer (HR = 1.05, 95% CI = 0.66–1.68).

**Conclusions:**

Patient and tumor characteristics predicted early‐stage CRC overtreatment. Overtreatment was associated with worse OS for colon but not rectal cancer.

## 1 Introduction

Although undertreating cancer may traditionally be more feared than overtreatment, there is a growing consensus on the harm caused by overtreating cancer [1, 2, 3, 4, 5, 6, 7, 8, 9]. Practice patterns are not uniform across different treatment centers: variations in patient characteristics, histologic assessment, and provider decision‐making can lead to treatment inconsistencies that subject certain patient populations to guideline‐discordant therapy with mixed clinical results [10, 11, 12, 13, 14, 15, 16, 17]. Age, sex, race, insurance status, and pre‐existing comorbidities have been identified as potential risk factors for guideline‐discordant treatment across different cancer types [18, 19, 20].

With over 150 000 Americans being diagnosed with colorectal cancer (CRC) annually [21], CRC overtreatment has the potential to harm a substantial number of patients. According to National Comprehensive Cancer Network (NCCN) guidelines, definitive surgery alone (colectomy for colon cancer and proctectomy with mesorectal excision for rectal cancer) is considered curative for cT1‐2N0M0 CRC [22, 23]. In the absence of pathologic upstaging, only observation is recommended, irrespective of the presence of high‐risk features [22, 23]. Any adjuvant therapy in this case poses greater potential risk (e.g., toxicity, decreased quality of life, anxiety, and financial burden) than benefit [24], constituting overtreatment [22, 23]. Studies have shown that CRC patients treated in accordance with NCCN guidelines have better outcomes than those with NCCN discordant treatment; most of these studies have primarily focused on cancer care delay or undertreatment [25, 26, 27, 28]. Less is known about the risk factors or the impact of overtreatment on CRC patients.

Despite some work assessing guideline‐discordant treatment of colon cancer a decade ago [19, 20], no recent multi‐institutional studies have comprehensively evaluated risk factors and survival outcomes associated with overtreatment in both colon and rectal cancer across all age groups [29]. This study addresses this gap by using the National Cancer Database (NCDB) to identify the predictors and overall survival (OS) impact of early‐stage CRC overtreatment, aiming to inform more evidence‐based and individualized treatment strategies, guidelines, and quality‐improvement initiatives. Specifically, this study aims to test our hypotheses that specific patient, tumor, and treatment facility characteristics are associated with an increased risk of overtreatment and that overtreatment negatively impacts OS.

## 2 Methods

### 2.1 Study Design and Population

This was a retrospective study from the NCDB that included patients aged 18–89 who underwent curative‐intent surgery between 2010 and 2020 across 1400 US hospitals, capturing about 70% of new cancer diagnoses in the United States [30, 31]. Cases included patients who underwent clinical stage T1‐T2N0M0 colon or rectal cancer resection. Since rectal cancer care exhibits nontrivial differences in treatment paradigms such as radiation, it was examined separately. Exclusion criteria included positive final surgical margins, preoperative systemic therapy or radiation, transanal or endoscopic excision, postoperative radiation for colon cancer, and missing data for any of the study variables. To avoid including patients who might have received therapy for recurrence rather than adjuvant intent as validated by previous work [32], patients who received adjuvant therapy more than 6 months after surgery were also excluded. The study was considered exempt from the IRB given the fully deidentified nature of the database. This study was conducted in accordance with the Strengthening the Reporting of Observational Studies in Epidemiology (STROBE) reporting guidelines.

### 2.2 Outcomes

**Figure 1:**
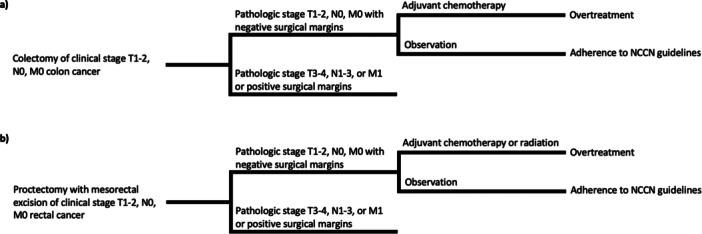
Overtreatment classification based on National Comprehensive Cancer Network (NCCN) guidelines for (a) colon and (b) rectal cancer.

Overtreatment was defined as receipt of adjuvant chemotherapy (or adjuvant radiation for rectal cancer). A detailed process for categorization of patients by overtreatment is presented in Figure 1. Only Stage I CRC patients who underwent definitive surgery (colectomy or proctectomy with mesorectal excision) were included, in accordance with NCCN guidelines deeming surgery alone curative for cT1‐2N0M0 disease regardless of high‐risk features. Exclusion of patients with non‐curative procedures and positive margins ensured consistent classification of overtreatment. As validated by previous NCDB studies [20], the primary outcome was the difference in OS (from date of diagnosis) between patients who were overtreated vs. patients who did not receive overtreatment. Secondary measures included risk factors associated with receipt of overtreatment.

### 2.3 Statistical Analyses

To compare cohorts by overtreatment and identify risk factors, we performed chi‐square and Fisher's exact tests for categorical variables as appropriate and Wilcoxon rank‐sum tests for continuous variables. *p* values were derived from two‐tailed tests, and significance was set a priori at *p* < 0.05. Univariable and multivariable logistic regressions were performed to assess if patient, hospital, and tumor characteristics were predictors of overtreatment. Covariates included age, sex, race, insurance, Charlson–Deyo Comorbidity Index, treatment facility type, top quartile facility case volume, histologic grade, histologic type, and clinical and pathologic T stage.

To create balanced samples for survival analyses, we created separate matched cohorts for colon and rectal cancer by overtreatment. Propensity matching with and without overtreatment (adjusting for the same covariates used in multivariable regression) was performed for colon and rectal cancer cases at a ratio of 10:1 to sufficiently power statistical analyses, which is a standard practice in the literature [33, 34]. A standardized mean difference (SMD) of < 0.15 was considered balanced [33]. Kaplan–Meier survival analyses with log‐rank tests as well as univariable and multivariable Cox proportional hazards analyses were performed to assess the association of overtreatment and matching variables with OS.

## 3 Results

After exclusion, the total colon cancer cohort included 22 875 cases, of which 144 (0.6%) received overtreatment (Table 1; Figure S1). The total rectal cancer cohort included 4198 cases, of which 82 (2.0%) received overtreatment. Among rectal cancer overtreatment cases, 21 (25.6%) involved chemotherapy only, 16 (19.5%) involved radiation only, and 45 (54.9%) involved both chemotherapy and radiation.

**Table 1:** Patient, facility, and tumor characteristics of colon and rectal cancer cases by overtreatment in the total cohort.

	Colon			Rectum		
Variable	No overtreatment	Overtreatment	*p*	No overtreatment	Overtreatment	*p*
Age, median [IQR]	69 [60‐77]	65.5 [57–72]	**< 0.001**	63 [54–71]	62.5 [53–72]	0.861
Sex			0.053			0.567
Male	11 261 (49.5%)	83 (57.6%)		2380 (57.8%)	50 (61.0%)	
Female	11,470 (50.5%)	61 (42.4%)		1736 (42.2%)	32 (39.0%)	
Race			**< 0.001**			0.576
White	19 406 (85.4%)	105 (72.9%)		3624 (88.0%)	72 (87.8%)	
Black	2403 (10.6%)	29 (20.1%)		263 (6.4%)	7 (8.5%)	
Other	922 (4.1%)	10 (6.9%)		229 (5.6%)	3 (3.7%)	
Insurance			**< 0.001**			**<0.001**
Uninsured	366 (1.6%)	8 (5.6%)		73 (1.8%)	42 (51.2%)	
Private/managed care	7748 (34.1%)	59 (41.0%)		2038 (49.5%)	7 (8.5%)	
Medicaid, Medicare, and other government	14 617 (64.3%)	77 (53.5%)		2005 (48.7%)	33 (40.2%)	
Above median income			**0.011**			0.459
0–47 999	8849 (38.9%)	71 (49.3%)		1493 (36.3%)	33 (40.2%)	
≥ 48 000	13 882 (61.1%)	73 (50.7%)		2623 (63.7%)	49 (59.8%)	
Charlson–Deyo Comorbidity Index			0.160			0.188
0	15 062 (66.3%)	103 (71.5%)		3004 (73.0%)	64 (78.0%)	
1	5143 (22.6%)	32 (22.2%)		788 (19.1%)	16 (19.5%)	
2+	2526 (11.1%)	9 (6.3%)		324 (7.9%)	2 (2.4%)	
Facility type			0.328			0.120
Non‐research/academic	16 866 (74.2%)	112 (77.8%)		2671 (64.9%)	60 (73.2%)	
Research/academic	5865 (25.8%)	32 (22.2%)		1445 (35.1%)	22 (26.8%)	
Top quartile facility case volume	5074 (22.3%)	26 (18.1%)	0.220	1199 (29.1%)	20 (24.4%)	0.349
Histologic grade			0.104			**< 0.001**
Well/moderately differentiated	20 891 (91.9%)	127 (88.2%)		3825 (92.9%)	68 (82.9%)	
Poorly/not differentiated	1840 (8.1%)	17 (11.8%)		291 (7.1%)	14 (17.1%)	
Histology			**0.001**			**0.009**
Nonmucinous adenocarcinoma	21 499 (94.6%)	131 (91.0%)		4054 (98.5%)	77 (93.9%)	
Mucinous adenocarcinoma	1152 (5.1%)	10 (6.9%)		57 (1.4%)	4 (4.9%)	
Signet ring cell carcinoma	80 (0.3%)	3 (2.1%)		5 (0.1%)	1 (1.2%)	
Clinical T stage			**0.001**			0.182
cT1	14 896 (65.5%)	76 (52.8%)		2063 (50.1%)	35 (42.7%)	
cT2	7835 (34.5%)	68 (47.2%)		2053 (49.9%)	47 (57.3%)	
Pathologic T stage			**0.009**			**< 0.001**
pT1	12 106 (53.3%)	61 (42.4%)		2149 (52.2%)	24 (29.3%)	
pT2	10 625 (46.7%)	83 (57.6%)		1967 (47.8%)	58 (70.7%)	
Adjuvant chemotherapy	0 (0%)	144 (100%)	NA	0 (0%)	66 (80.5%)	NA
Adjuvant radiation	—	—	NA	0 (0%)	61 (74.4%)	NA

*Note:* Values significant at *p* < 0.05 are bolded.

Abbreviations: IQR, interquartile range; NA, not applicable.

On multivariable logistic regression (Table 2), overtreatment of colon cancer was associated with younger age (aOR = 0.96, 95% CI = 0.95–0.98, *p* < 0.001), Black race vs. White (aOR = 1.94, 95% CI = 1.26–2.99, *p* = 0.002), race other than White or Black (aOR = 2.04, 95% CI = 1.06–3.95, *p* = 0.034), and pathologic Stage T2 vs. T1 (aOR = 1.66, 95% CI = 1.19–2.33, *p* = 0.003). Overtreatment was not associated with female sex (aOR = 0.73, 95% CI = 0.52–1.02, *p* = 0.063), above median income (aOR = 0.71, 95% CI = 0.51–1.00, *p* = 0.050), poor/undifferentiated grade (aOR = 1.58, 95% CI = 0.95–2.64, *p* = 0.080), and high‐risk histology (aOR = 1.76, 95% CI = 0.98–3.15, *p* = 0.057). Overtreatment of rectal cancer was associated with pathologic Stage T2 vs. T1 (aOR = 2.58, 95% CI = 1.59–4.19, *p* < 0.001), poor/undifferentiated grade (aOR = 2.61, 95% CI = 1.44–4.76, *p* = 0.002), and high‐risk histology (aOR = 3.20, 95% CI = 1.22–8.40, *p* = 0.018).

**Table 2:** Univariable and multivariable logistic regressions of matching variables by overtreatment.

	Colon								Rectum							
	Univariable				Multivariable				Univariable				Multivariable			
Variable	OR (95% CI)		*p*		aOR (95% CI)		*p*		OR (95% CI)		*p*		aOR (95% CI)		*p*	
Age (1 year increase)	0.97 (0.95–0.98)		< 0.001		**0.96 (0.95–0.98)**		**< 0.001**		1.00 (0.98–1.02)		0.902		1.00 (0.97–1.02)		0.770	
Female sex (vs. male)	0.72 (0.52–1.01)		0.054		0.73 (0.52–1.02)		0.063		0.88 (0.56–1.37)		0.567		0.88 (0.56‐1.38)		0.565	
Race (vs. White)																
Black	2.23 (1.48–3.37)		< 0.001		**1.94 (1.26–2.99)**		**0.002**		1.34 (0.61–2.94)		0.466		1.35 (0.60–3.01)		0.471	
Other	2.00 (1.04–3.85)		0.037		**2.04 (1.06–3.95)**		**0.034**		0.66 (0.21–2.11)		0.483		0.75 (0.23–2.42)		0.630	
Private insurance	1.34 (0.96–1.87)		0.083		0.87 (0.58–1.30)		0.488		1.07 (0.69–1.66)		0.760		1.10 (0.62–1.95)		0.743	
Above median income	0.66 (0.47–0.91)		0.012		0.71 (0.51–1.00)		0.050		0.85 (0.54–1.32)		0.460		0.83 (0.53–1.33)		0.443	
Any comorbidity (vs. no comorbidity)	0.78 (0.54–1.12)		0.184		0.83 (0.57–1.20)		0.311		0.76 (0.45–1.29)		0.307		0.71 (0.41–1.23)		0.225	
Research/academic facility	0.82 (0.55–1.22)		0.328		0.78 (0.51–1.19)		0.246		0.68 (0.41–1.11)		0.122		0.69 (0.40–1.20)		0.194	
Top quartile facility case volume	0.77 (0.50–1.17)		0.221		0.80 (0.51–1.25)		0.325		0.78 (0.47–1.30)		0.350		0.95 (0.54–1.68)		0.857	
Poorly differentiated/undifferentiated (vs. well/moderately differentiated)	1.52 (0.91–2.53)		0.107		1.58 (0.95–2.64)		0.080		2.71 (1.50–4.87)		0.001		**2.61 (1.44–4.76)**		**0.002**	
High‐risk histology (vs. nonmucinous adenocarcinoma)	1.73 (0.98–3.07)		0.060		1.76 (0.98–3.15)		0.057		4.25 (1.66–10.86)		0.003		**3.20 (1.22–8.40)**		**0.018**	
pT2 (vs. pT1)	1.55 (1.11–2.16)		0.010		**1.66 (1.19–2.33)**		**0.003**		2.64 (1.63–4.27)		< 0.001		**2.58 (1.59–4.19)**		**< 0.001**	

*Note:* Multivariable values significant at *p* < 0.05 are bolded.

Abbreviations: aOR, adjusted odds ratio; CI, confidence interval; OR, odds ratio.

**Figure 2:**
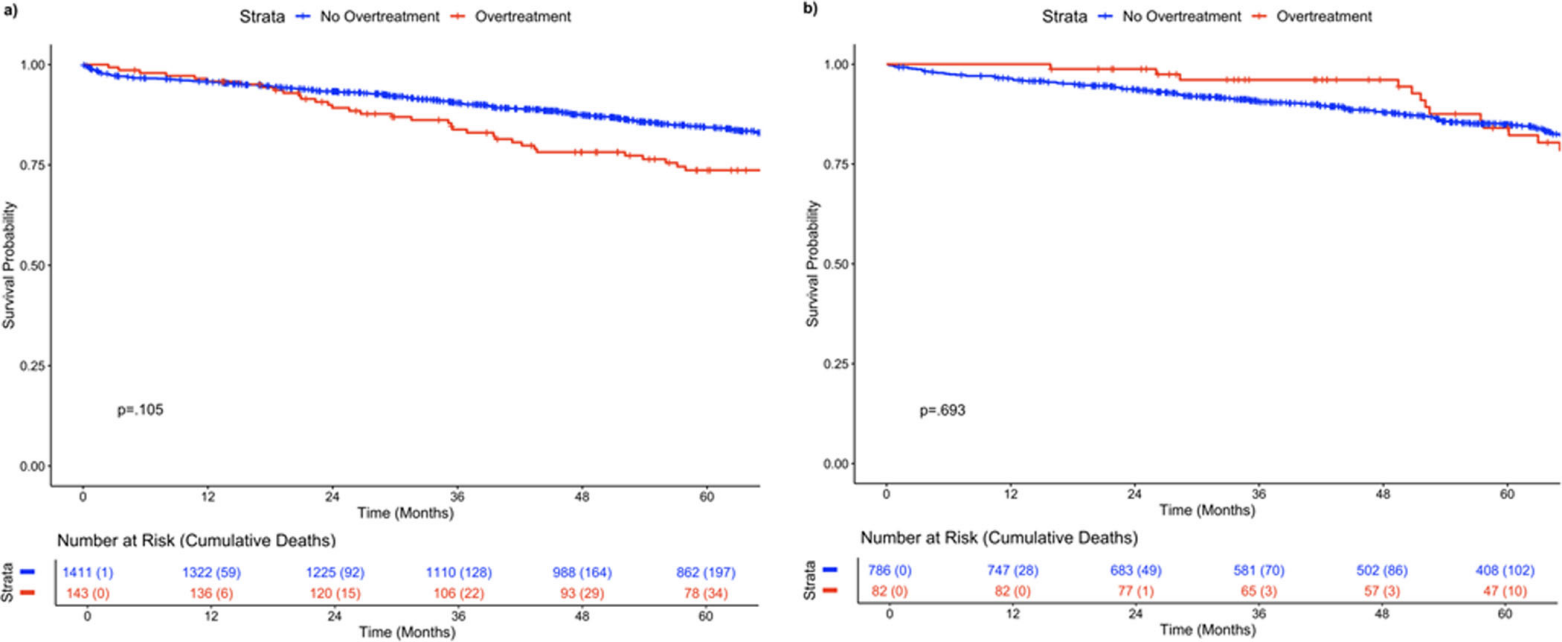
Kaplan–Meier survival analyses by overtreatment for (a) colon and (b) rectal cancer.

The propensity‐matched cohorts included 144 overtreatment and 1418 non‐overtreatment colon cancer cases as well as 82 overtreatment and 786 non‐overtreatment rectal cancer cases (Table S1). All SMDs were less than 0.15, indicating appropriate balance (Table S2). Colon cancer overtreatment exhibited a nonsignificant trend toward worse OS on Kaplan–Meier survival analysis (Figure 2; *p* = 0.105). Rectal cancer overtreatment was not associated with an OS difference on Kaplan–Meier survival analysis (*p* = 0.693). On Cox proportional hazard analysis (Table 3), overtreatment for colon cancer was independently associated with worse OS (HR = 1.40, 95% CI = 1.01–1.93, *p* = 0.042) after controlling for propensity‐matched variables. This indicates a 40% increased hazard of death. Overtreatment for rectal cancer was not independently associated with an OS difference (HR = 1.05, 95% CI = 0.66–1.68, *p* = 0.844).

**Table 3:** Univariable and multivariable Cox proportional hazard analyses of overtreatment on overall survival.

	Colon				Rectum			
	Univariable		Multivariable		Univariable		Multivariable	
Variable	HR (95% CI)	*p*	HR (95% CI)	*p*	HR (95% CI)	*p*	HR (95% CI)	*p*
Age (1 year increase)	1.07 (1.05–1.08)	< 0.001	**1.06 (1.05–1.07)**	**< 0.001**	1.07 (1.06–1.09)	< 0.001	**1.07 (1.05–1.09)**	**< 0.001**
Female sex (vs. male)	0.89 (0.72–1.10)	0.285	**0.76 (0.61–0.95)**	**0.014**	0.89 (0.65–1.22)	0.468	0.85 (0.62–1.18)	0.336
Race (vs. White)								
Black	1.02 (0.78–1.32)	0.902	**1.35 (1.02–1.79)**	**0.034**	1.52 (0.95–2.45)	0.084	**1.86 (1.14–3.05)**	**0.014**
Other	0.53 (0.30–0.94)	0.031	0.70 (0.39–1.25)	0.230	0.61 (0.23–1.66)	0.337	0.60 (0.22–1.64)	0.318
Private insurance	0.35 (0.27–0.45)	< 0.001	0.75 (0.57–1.00)	0.052	0.33 (0.24–0.46)	< 0.001	0.91 (0.61–1.37)	0.664
Above median income	0.86 (0.70–1.06)	0.148	0.91 (0.73–1.14)	0.423	0.76 (0.57–1.03)	0.074	0.76 (0.56–1.03)	0.073
Any comorbidity (vs. no comorbidity)	2.19 (1.78–2.69)	< 0.001	**1.71 (1.38–2.12)**	**< 0.001**	1.92 (1.41–2.61)	< 0.001	**1.47 (1.06–2.02)**	**0.019**
Research/academic facility	1.04 (0.81–1.35)	0.749	**1.33 (1.02–1.74)**	**0.037**	0.54 (0.37–0.80)	0.002	0.73 (0.47–1.16)	0.182
Top quartile facility case volume	0.86 (0.64–1.14)	0.286	0.76 (0.56–1.02)	0.071	0.58 (0.38–0.87)	0.008	0.87 (0.54–1.40)	0.556
Poorly differentiated/undifferentiated (vs. well/moderately differentiated)	1.00 (0.72–1.39)	0.997	0.91 (0.65–1.27)	0.587	1.13 (0.74–1.72)	0.580	1.02 (0.66–1.57)	0.934
High‐risk histology (vs. nonmucinous adenocarcinoma)	1.42 (0.98–2.07)	0.065	1.21 (0.82–1.78)	0.338	1.85 (0.95–3.62)	0.072	**2.20 (1.10–4.40)**	**0.025**
pT2 (vs. pT1)	1.19 (0.97–1.47)	0.101	1.01 (0.81–1.25)	0.954	1.40 (0.97–2.01)	0.070	1.14 (0.79–1.66)	0.480
Overtreatment	1.30 (0.94–1.80)	0.107	**1.40 (1.01–1.93)**	**0.042**	1.10 (0.69–1.75)	0.694	1.05 (0.66–1.68)	0.844

*Note:* Multivariable values significant at *p* < 0.05 are bolded.

Abbreviations: CI, confidence interval; HR, hazard ratio.

## 4 Discussion

This study shows that overtreatment of early‐stage colon and rectal cancer is rare, occurring in only 0.6% and 2.0% of cases, respectively. However, overtreatment is associated with distinct demographic and tumor characteristics. For colon cancer, younger age, Black and other non‐White race, and pathologic Stage T2 (compared to pT1) are significant predictors of overtreatment, with nonsignificant trends suggesting associations with poor/undifferentiated grade, high‐risk histology, lower income, and male sex. For rectal cancer, overtreatment is associated with pathologic Stage T2, poor/undifferentiated grade, and high‐risk histology. Importantly, overtreatment of colon cancer is independently associated with worse OS, while no significant OS difference is observed for rectal cancer overtreatment.

Several studies have examined the factors leading to overtreatment of different types of cancer. For example, Papaleontiou et al. [35] found that case volume played a significant role in the overtreatment of low‐risk thyroid cancer with radioactive iodine (RAI). Pak et al. [36] and others explored overtreatment at various stages of breast cancer management. Howard et al. [18] identified racial, sexual, and insurance status differences in the over‐ and under‐treatment of renal cancers. The demographic variables identified in our study align with those highlighted by Howard et al. [18] in the treatment of renal cancers.

In addition to other types of cancer, some demographic variables have been examined in NCCN‐discordant colon cancer treatment. Previous work on colon cancer showed that adults under 50 years old with more comorbidities were significantly more likely to receive adjuvant chemotherapy compared to older patients, with a marginal improvement in survival [20]. Our study validates these results, as we also find that overtreatment is associated with younger age in colon cancer patients. While Kneuertz et al. did not have enough patients to analyze Stage I colon cancer, the study found no statistically significant difference in survival for Stage II colon cancer treated with adjuvant chemotherapy [20]. Using less stringent exclusion criteria for 2003–2007 data, Chagpar et al. [19] found an overtreatment rate of 2.8% as well as an association between age, race/ethnicity, and insurance status with NCCN‐discordant treatment of Stage I colon cancer. We similarly found that age, race, and pathologic T stage were significant predictors for a patient receiving overtreatment for colon cancer. Given that young‐onset colon cancer is associated with more advanced disease at diagnosis [37] and that Black patients generally experience worse outcomes in colon cancer [38, 39], we hypothesize that these patients are treated more aggressively and are more likely to receive NCCN‐discordant regimens. We also believe the lower rate of Stage I colon cancer overtreatment (0.6%) from 2010 to 2020 may be due to changed NCCN guidelines backed by substantially more literature.

This study found a higher rate of overtreatment for rectal cancer compared to colon cancer. Colon and rectal cancers, while often grouped under the umbrella of CRC, exhibit distinct tumor biology and are typically managed with different therapeutic protocols. From a biological perspective, rectal cancers are more likely to exhibit higher rates of microsatellite stability, and a distinct tumor microenvironment compared to colon cancers [40, 41]. Additionally, rectal tumors tend to have a higher propensity for local recurrence, which often justifies the use of neoadjuvant radiation therapy [42]. Surgery for rectal cancer is significantly more challenging with a higher risk of morbidity [43]. Along with biology, management also differs, with colon cancer treated with surgery followed by observation or adjuvant chemotherapy based on pathologic staging and high‐risk features [44]. Conversely, rectal cancer often involves a multimodal approach, including neoadjuvant chemoradiation followed by surgery, and sometimes adjuvant chemotherapy depending on the clinical and pathological stage. Accordingly, the addition of adjuvant therapy in early‐stage rectal cancer, particularly when not clinically indicated, may be less impactful on OS due to prior exposure to neoadjuvant treatments or may reflect more nuanced clinical decision‐making. These inherent differences in biology and treatment strategies may help explain why overtreatment was associated with worse OS in colon cancer but not in rectal cancer in our study.

Compared to previous work, our study is unique in that we used NCDB data through 2020 and had a large enough cohort to assess a wider range of potential risk factors for overtreatment among patients with Stage I colon cancer. Moreover, we performed propensity matching for several demographic and oncologic variables to control for covariates and assessed the OS impact of overtreatment in Stage I colon cancer. Additionally, our study not only added novel insights to prior research on colon cancer overtreatment but also analyzed rectal cancer, which was twice as prevalent as colon cancer. This discrepancy may be due to differences in biology and treatment strategy.

## 5 Limitations

This NCDB study exhibits some limitations. One potential limitation is the relatively small number of patients receiving overtreatment compared to the total cohort. Specifically, there were 144 and 81 overtreatment cases for colon and rectal cancer, respectively, out of tens of thousands of CRC cases without overtreatment. This discrepancy could introduce sampling bias and potentially limit the identification of overtreatment risk factors. Nevertheless, we used multivariable analysis and propensity matching to mitigate this concern. The sample sizes were sufficiently large to generate narrow confidence intervals, supporting the validity of our analysis. Moreover, our findings exhibit a discrepancy in that overtreatment of colon cancer was independently associated with worse OS in the adjusted Cox proportional hazards model (*p* = 0.042), whereas the unadjusted Kaplan–Meier analysis showed only a nonsignificant trend toward worse survival (*p* = 0.105), which suggests the observed survival difference may be influenced by statistical adjustments. One possible explanation is that confounding variables controlled for in the Cox model, such as demographic and oncologic factors, help reveal an association that is otherwise obscured in the unadjusted analysis. Additionally, the relatively small number of overtreatment cases may contribute to model instability that results in borderline statistical significance near the *p* < 0.05 threshold. External validation in independent patient cohorts would help determine if the discrepancy is due to model instability or an obscured association on univariable analysis that would be cleared in a specific subpopulation. Additionally, NCDB provides limited data on variables related to surgical outcomes, quality of life, or other patient‐centered outcomes. We are unable to determine the effect of overtreatment on colorectal‐specific adverse events (e.g., perforation or colitis). Similarly, NCDB might not include all variables that drive adjuvant therapy decisions. Unmeasured factors such as patient performance status, physician judgment, and individual patient preferences are not captured in the NCDB. These variables likely affect both treatment selection and subsequent outcomes, introducing the potential for residual confounding. Without accounting for these factors, our findings may be influenced by unmeasured selection biases that could either overestimate or underestimate the impact of overtreatment on survival. However, NCDB's strengths include its large patient population (capturing about 70% of cancer cases [31]) and extensive cancer treatment data, which have been leveraged in numerous CRC studies [10, 14, 19, 20, 33, 45, 46, 47]. Future research incorporating more granular clinical variables, patient‐reported outcomes, and prospective data collection would provide a more comprehensive understanding of how overtreatment impacts long‐term patient outcomes.

## 6 Conclusions

In this large NCDB analysis, overtreatment of early‐stage CRC was uncommon yet clinically significant. While only a small proportion of patients with colon (0.6%) and rectal (2.0%) cancer received overtreatment (i.e., adjuvant therapy despite NCCN guideline recommendations for surgery alone), certain demographic (e.g., younger age, non‐White race) and tumor‐related (e.g., higher T stage and poor differentiation) factors were associated with increased odds of overtreatment. Overtreatment was significantly associated with worse OS in colon cancer but not rectal cancer, suggesting potential differences in treatment paradigms or disease biology. While undertreatment of cancers can be harmful, these findings highlight the importance of physicians' understanding of NCCN guidelines and caution against adjuvant therapy overuse, which will help minimize systemic toxicity and improve outcomes, especially in certain patient populations. Factors regarding the institution (e.g., institutional guidelines), physician (e.g., experience with disease pathology), and patient (e.g., preference) should be teased out to understand their impact on the administration of adjuvant treatment and clinical outcomes. Understanding the drivers of overtreatment can help implement system‐level interventions to promote both guideline‐concordant and individualized care.

## Conflicts of Interest

The authors declare no conflicts of interest.

## Synopsis

This National Cancer Database study found that 0.6% and 2.0% of cT1‐2N0M0 colon and rectal cancer cases, respectively, receive adjuvant therapy after surgery, constituting overtreatment. Colon cancer overtreatment was associated with younger age, Black race, and pathologic stage T2 (vs pT1), while rectal cancer overtreatment was associated with pT2, poor/undifferentiated grade, and high‐risk histology. Overtreatment was associated with worse overall survival for colon but not rectal cancer.

## Supporting information

Supplement.

## Data Availability

Data sharing is not applicable to this article as no new data were created or analyzed in this study.
