# Supplementary material for: Less Is More: Risk Factors and Survival Outcomes of Overtreatment for Early‐Stage Colorectal Cancer
Source: J Surg Oncol. 2025 Jul 5;132(3):427–36. doi: 10.1002/jso.70028 (PMC12455544; doi:10.1002/jso.70028)
Supplement: Supplementary file 1 — Supplement. [file JSO-132-427-s001.docx]

**Supplemental Figure 1**. Study Schemata for (a) Colon and (b) Rectal Cancer


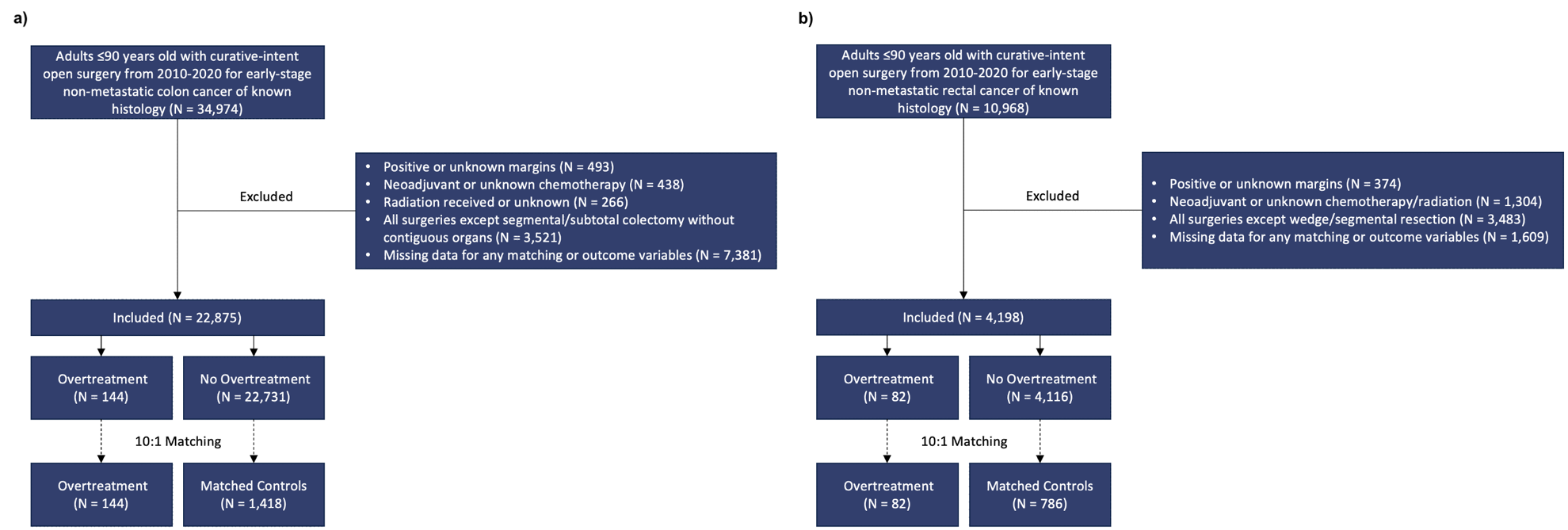


**Supplemental Table 1.** Patient, Facility, and Tumor Characteristics of Colon and Rectal Cancer Cases by Overtreatment in the Matched Cohort

|  | **Colon** | | | **Rectum** | | |
| --- | --- | --- | --- | --- | --- | --- |
| **Variable** | **No Overtreatment** | **Overtreatment** | **P Value** | **No Overtreatment** | **Overtreatment** | **P Value** |
| **Age, median [IQR]** | 65 [56-73] | 65.5 [57-72] | .823 | 63 [54.25-72] | 62.5 [53-72] | .596 |
| **Sex** |  |  | .240 |  |  | .311 |
| Male | 888 (62.6%) | 83 (57.6%) |  | 523 (66.5%) | 50 (61.0%) |  |
| Female | 530 (37.4%) | 61 (42.4%) |  | 263 (33.5%) | 32 (39.0%) |  |
| **Race** |  |  | .933 |  |  | .896 |
| White | 1054 (74.3%) | 105 (72.9%) |  | 690 (87.8%) | 72 (87.8%) |  |
| Black | 272 (19.2%) | 29 (20.1%) |  | 60 (7.6%) | 7 (8.5%) |  |
| Other | 92 (6.5%) | 10 (6.9%) |  | 36 (4.6%) | 3 (3.7%) |  |
| **Insurance** |  |  | .917 |  |  | .894 |
| Uninsured | 73 (5.1%) | 8 (5.6%) |  | 399 (50.8%) | 42 (51.2%) |  |
| Private/Managed Care | 595 (42.0%) | 59 (41.0%) |  | 57 (7.3%) | 7 (8.5%) |  |
| Medicaid, Medicare, Other  Government | 750 (52.9%) | 77 (53.5%) |  | 330 (42.0%) | 33 (40.2%) |  |
| **Above Median income** |  |  | .947 |  |  | .917 |
| 0-47,9999 | 695 (49.0%) | 71 (49.3%) |  | 321 (40.8%) | 33 (40.2%) |  |
| ≥48,000 | 723 (51.0%) | 73 (50.7%) |  | 465 (59.2%) | 49 (59.8%) |  |
| **Charlson-Deyo Comorbidity Index** |  |  | .935 |  |  | .893 |
| 0 | 995 (70.2%) | 103 (71.5%) |  | 595 (75.7%) | 64 (78.0%) |  |
| 1 | 326 (23.0%) | 32 (22.2%) |  | 169 (21.5%) | 16 (19.5%) |  |
| 2+ | 97 (6.8%) | 9 (6.3%) |  | 22 (2.8%) | 2 (2.4%) |  |
| **Facility type** |  |  | .519 |  |  | .805 |
| Non-Research/Academic | 1135 (80.0%) | 112 (77.8%) |  | 565 (71.9%) | 60 (73.2%) |  |
| Research/Academic | 283 (20.0%) | 32 (22.2%) |  | 221 (28.1%) | 22 (26.8%) |  |
| **Top Quartile Facility Case Volume** | 257 (18.1%) | 26 (18.1%) | .984 | 205 (26.1%) | 20 (24.4%) | .739 |
| **Histologic Grade** |  |  | .689 |  |  | .208 |
| Well/Moderately Differentiated | 1266 (89.3%) | 127 (88.2%) |  | 690 (87.8%) | 68 (82.9%) |  |
| Poorly/Not Differentiated | 152 (10.7%) | 17 (11.8%) |  | 96 (12.2%) | 14 (17.1%) |  |
| **Histology*** |  |  | .190 |  |  | .164 |
| Nonmucinous Adenocarcinoma | 1330 (93.8%) | 131 (91.0%) |  | 765 (97.3%) | 77 (93.9%) |  |
| High-Risk Histology | 88 (6.2%) | 13 (9.0%) |  | 21 (2.7%) | 5 (6.1%) |  |
| **Clinical T stage** |  |  | .956 |  |  | .730 |
| cT1 | 745 (52.5%) | 76 (52.8%) |  | 320 (40.7%) | 35 (42.7%) |  |
| cT2 | 673 (47.5%) | 68 (47.2%) |  | 466 (59.3%) | 47 (57.3%) |  |
| **Pathologic T stage** |  |  | .803 |  |  | .620 |
| pT1 | 616 (43.4%) | 61 (42.4%) |  | 210 (26.7%) | 24 (29.3%) |  |
| pT2 | 802 (56.6%) | 83 (57.6%) |  | 576 (73.3%) | 58 (70.7%) |  |
| **Adjuvant Chemotherapy** | 0 (0%) | 144 (100%) | *NA* | 0 (0%) | 66 (80.5%) | *NA* |
| **Adjuvant Radiation** | - | - | *NA* | 0 (0%) | 61 (74.4%) | *NA* |

*Note: Due to the small number of signet ring cell carcinoma cases, mucinous adenocarcinoma and signet ring cell carcinoma cases were combined into a high-risk histology group for statistical comparison.

IQR: interquartile range; NA: not applicable

Values significant at p<.05 are bolded.

**Supplemental Table 2.** Covariate Balance Before and After Propensity Matching

|  | **Colon** | | **Rectum** | |
| --- | --- | --- | --- | --- |
| **Variable** | **Pre-Matching SMD** | **Post-Matching SMD** | **Pre-Matching SMD** | **Post-Matching SMD** |
| Age | -0.38 | -0.02 | -0.01 | -0.06 |
| Female Sex (vs Male) | -0.16 | 0.10 | -0.06 | 0.12 |
| Race |  |  |  |  |
| White | -0.28 | -0.02 | -0.01 | -0.01 |
| Black | 0.24 | 0.01 | 0.08 | 0.04 |
| Other | 0.11 | 0.02 | -0.10 | -0.04 |
| Insurance |  |  |  |  |
| Uninsured | 0.17 | 0.00 | -0.14 | 0.00 |
| Private/Managed Care | 0.14 | -0.02 | 0.03 | 0.04 |
| Medicaid | 0.06 | 0.05 | 0.12 | -0.06 |
| Medicare/Other Government | -0.24 | 0.00 | -0.07 | -0.01 |
| Income Above Median | -0.21 | 0.00 | -0.08 | 0.03 |
| Comorbidity Index |  |  |  |  |
| 0 | 0.12 | 0.03 | 0.12 | 0.06 |
| 1 | -0.01 | -0.02 | 0.01 | -0.06 |
| 2+ | -0.20 | -0.02 | -0.35 | -0.02 |
| Research/Academic Facility | -0.09 | 0.04 | -0.19 | -0.01 |
| Top Quartile Facility Case Volume | -0.11 | -0.01 | -0.11 | -0.03 |
| Poorly Differentiated/Undifferentiated | 0.12 | 0.01 | 0.27 | 0.10 |
| Histology |  |  |  |  |
| Nonmucinous Adenocarcinoma | -0.13 | -0.07 | -0.19 | 0.01 |
| Mucinous Adenocarcinoma | 0.07 | 0.09 | 0.16 | -0.06 |
| Signet Ring Cell Carcinoma | 0.12 | -0.01 | 0.10 | 0.11 |
| cT2 (vs cT1) | 0.26 | -0.01 | 0.15 | -0.04 |
| pT2 (vs pT1) | 0.22 | 0.02 | 0.50 | -0.08 |

SMD: Standardized mean difference
